# Supplementary material for: Expression analysis of p16 and TOP2A protein biomarkers in cervical cancer lesions and their correlation with clinico-histopathological characteristics in a referral hospital, Tanzania
Source: PLoS One. 2021 Oct 27;16(10):e0259096. doi: 10.1371/journal.pone.0259096 (PMC8550370; doi:10.1371/journal.pone.0259096)
Supplement: Attachment — Submitted filename: Response to Reviewers.docx [file pone.0259096.s003.docx]

**01^st^ August, 2021**

**Response to reviewers’ comments:**

**Ref**: PONE-D-21-05111R1

**Title**: Expressions of p16 and TOP2A protein biomarkers in cervical cancer lesions and their correlation with clinico-histopathological characteristics in Tanzania

**Journal**: PLOS ONE

Dear Editor,

Thank you very much for giving us an opportunity to revise our manuscript. We very much appreciate your reviewers for the positive and constructive comments on our manuscript. Based on comments we received, careful modifications have been made to the manuscript as will be seen in the resubmitted version of this manuscript. Significant changes have been made throughout the manuscript for the utility of the studied biomarkers for diagnosis rather than screening purposes. In addition, the introduction has been revised in clearly defining the study goal. Conclusion, data analysis and interpretation have been thoroughly revised and rewritten as recommended by editorial and reviewers’ comments. All changes are in track changes mode. We hope the revised manuscript will meet the standards for the PLoS ONE journal. Please, also find our point-by-point responses to the raised queries/comments. The revised manuscript has been resubmitted. Please address any correspondence regarding the manuscript to me directly. We look forward to your positive response.

Sincerely,

Zavuga Zuberi and authors

**Editors’ Comments**

While the manuscript was significantly improved, one critical issue remains. Namely, the authors use biopsy material throughout the study. Biopsy material should not be considered a part of the cervical screening process. On the other hand, the authors give a lot of emphasis on the usability of the tested markers in the screening despite not testing them in the screening setting (i.e. lines 52, 64-65, 106, 109, 341, 352). The manuscript should be revised so that it is evident that results obtained on cervical biopsies are not in fact intended for screening purposes and that cervical cancer screening is unlikely to ever include imunohistochemical marker staining of cervical biopsies as routine. Referring everyone for biopsy is not a good screening strategy. Also, the authors imply their study goal was to address the lack of „low-cost, sensitive and specific screening methods... in Tanzania. Therefore…“ (line 106). The data obtained on (mostly) cervical cancer biopsies only implies that those markers might also work well in a screening setting but the current manuscript cannot provide solid evidence to that end.

Response: Thank you for your comment. The usefulness of immunohistochemistry assay in improving diagnosis of cervical cancer lesions has been considered throughout the manuscript. For instance, P4L67-69, P5L84-85, P19L317, P20L349-351, etc.

On the other hand, the authors fail to significantly explore the utility of having p16/TOP2A results on the diagnosis and/or prognosis of cervical cancer in Tanzania despite this being mentioned (lines 101, 105, 109). For example, would having those results clarify the „Non-definitive diagnosis” cases, how common are “non-definitive diagnosis” cases overall and would additional biomarker results affect treatment and possibly prognosis/survival of those or all patients?

Response: Thank you for your comment. “Non-definitive diagnosis” was used to refer to the preliminary H&E results of the cervical biopsy. Based on the relatively low number (n=6) of non-definitive cases, they were excluded in the statistical analyses for the correlation, diagnostic performance and strength of associations as they may confer very minimal risk of cervical cancer development.

Overall, while the methods are clearly described and the experiments conducted adequately, the conclusions and the interpretation of the data is not appropriate ([Publication criteria 4](https://journals.plos.org/plosone/s/criteria-for-publication)) and needs to be revised, expanded or at least rephrased extensively. Screening should be de-emphasized throughout the manuscript and only briefly mentioned as a possible use of those markers since the presented data doesnt address this issue.

Response: Thank you for your useful comments. Our conclusion and data interpretation have been re-written to de-emphasize about screening throughout the manuscript. The conclusion is now revised in a way that supports the data presented as highlighted in the publication criteria 4 (P22L374-380).

**Additional Editor Comments**

Typo: L80 „frequentky”

Response: Thank you for your comment. Typing error has been corrected (P5L85).

L159 How does

Response: Thank you for your comment. Unfortunately, the concern raised is incomplete.

Software error L202 „(Table 1Error! Reference source not found.).”

Response: Thank you for your comment. The error associated with document formatting has been corrected (P11L225).

Software error L208, L212, 214, 216,249 as above

Response: Thank you for your comment. All the software errors in the document have been corrected (P12L234, P12L236, P12L237, P16L272).

L275 sentence unclear „Nevertheless, cancerous cervical lesions were significantly regressed with the expression of p16 than non-cancerous cervical lesions“

Response: Thank you for your good observation. The sentence has been revised to “Nevertheless, cancerous cervical lesions were statistically correlated with p16 expression” (P16L272).

Typo L338 "p1"

Response: Thank you for your comment. Typing error has been corrected (P21L356).

L258, instead of coding Benign lesions as 1 and all other lesions as 0 for the Table 3 correlations, it might be better to simply code disease stages as 0 for benign, 1 for precancer and 2 as cancer and calculate the correlation with this one parameter instead of 3 different binary parameters for a single variable (histopathological class).

Response: Thank you for your good observation. The data for correlation test were recoded for disease stages as “0” for benign, “1” for precancerous and “2” for cancerous. Moreover, data inference was revised as per the calculated correlation output (Table 3 and P16L263-274).

**Reviewer #2**

Reviewer #2: Although the manuscript was somehow improved the authors did not address all my comments raised in a previous round of review.

Response: Thank you for your comment. However, substantial changes have been made to improve the manuscript as will be reviewed.

Major deficiency of the study to resolve

As mentioned in my previous review, the clear goal of the study would be desirable taking into consideration the basic knowledge on CC screening and diagnostic procedures. The study needs to clearly distinguish the screening test from confirmation testing, that is the case in this study on archival biopsies. HPV test results predict the risk of cervical cancer and its precursors (cervical intraepithelial neoplasia) better than cytological or colposcopic/visual abnormalities, which indicate only signs of HPV infection.

Response: Thank you for your comment. Yes, we agree with the reviewer, this study basically was not intended to assess the screening tests for cervical cancer, rather we conceived the present study to evaluate the usefulness of p16 and TOP2A as potential biomarkers in dysplastic and malignant alteration of cervical epithelium by analyzing a series of benign, precancerous and cancerous cervical lesions so as to assess whether their expression might be of any use in predicting prognosis in cervical carcinogenesis (P6L130-134).

The results of HPV testing would add value to the study as the limitation of the study is a relatively low number of analysed samples (n=145). Therefore, the authors are advised to provide those results.

Response: Thank you for a very useful comment. However, due to limited financial support received as the part of Master’s studies, genotyping of HPV subtypes using archived cervical biopsies was not performed.

We kindly acknowledge the importance of genotyping HPV subtypes from the archived cervical biopsies in predicting the risks associated with the development of cervical cancer lesions and its precursors that may provide a different pattern in association with the expression levels of p16 and TOP2A biomarkers. This information has been updated in the manuscript (P21L369-373). This information has been taken care by recommending genotyping of HPV subtypes in a further study.

It would be interesting to refer to the crude and effective cervical cancer screening coverage for women aged 25 to 64 years in Tanzania in the Introduction.

Response: Thank you for your comment. “In Tanzania, very limited studies estimating the coverage of cervical cancer screening by age exist [12]. However, a follow-up study that enrolled women aged between 25 and 60 years from three cervical cancer screening clinics from urban and semi-rural areas in Tanzania reported 17.2% (696/4,043) and 14.2% (438/3,074) of the women aged between 25 and 60 years had high-risk HPV (hr-HPV) at baseline and in the first follow-up, respectively [13]. Only 3.4% (139/4,043) of the women aged between 25 and 60 years had high-grade squamous intraepithelial lesion (HSIL) in the same study [13]” (P5L93-99).

Major remarks

L80-81 delete “These methods have poor sensitivity and specificity and are very subjective.” This statement is inappropriate.

Response: Thank you for your good comment. The statement ‘These methods have poor sensitivity and specificity and are very subjective’ has been deleted’.

In L77 the authors are saying that “there are inadequate immunohistopathological testing facilities”, which explains why the most common screening CC test (Pap test) is not implemented in Tanzania. The authors can say, of cause, if it is the case “These methods require well-educated and qualified staff as well as high quality control in test implementation, that, unfortunately is not provided in Tanzania”.

Response: Thank you for your good comment. The sentence ‘there are inadequate immunohistopathological testing facilities’ has been deleted and replaced with the sentence you have advised (P5L81-83). However, Pap test is available in the zonal hospitals in Tanzania (P5L91-92).

L81-83 “According to Dartell et al. [11] reported lower sensitivity of visual inspection with acetic acid than HPV-testing in detecting HSIL cervical lesions during a hospital-based cervical cancer screening event in Dar es Salaam, Tanzania.” Indeed, HPV-testing as primary screening is considered a gold standard in developing countries, especially because of the lack of good cytology facilities. My advice to authors is to check the worldwide literature on CC screening programs, especially those published and acknowledged by IARC and WHO. Thus, complete the paragraph with the reasoning leading to the adequate use of cellular biomarkers in this study.

Response: Thank you for your good comment. The paragraph has been revised to “Another study by Dartell et al. [11] reported lower sensitivity of VIA than HPV-testing in detecting HSIL cervical lesions during a hospital-based cervical cancer screening event in Dar es Salaam, Tanzania. In many high-income countries, cervical cancer rates declined substantially after the wide spread introduction of the Pap test in the mid 20^th^ century [12]. However, similar trends did not emerge in low- and middle-income countries including Tanzania due to lack of resources for screening implementation including cytology review and low population coverage leading to advanced detection of cervical cancer and poor survival rates [2, 12]” (P5L99-106).

L86, L98, L100 These immunohistological test are not appropriate for screening, although useful to improve diagnostic of CC on biopsy were cytology fails. Thus, reconsider this phrase and the whole purpose of your study.

Response: Thank you for your comment. The usefulness of immunohistochemistry assay in improving diagnosis of cervical cancer lesions has been considered throughout the manuscript. For instance, P6L109, P6L121, P6L123, etc.

L106 Pay attention to the use of cheap HPV tests design for the developing countries rather that immunohistological test, which are not cheap at all.

Response: Thank you for your very useful comment. The sentence has been revised to capture low-cost HPV DNA tests such as *care*HPV (P7L128-130).

L109-L110. I am not convinced of the screening use of the mentioned biomarkers. So, reconsider the purpose of your study.

Response: Thank you for your comment. This has been re-written throughout the manuscript and the markers herein were not studied for screening but rather as future possible diagnostic markers.

Minor corrections to do

L80 delete “Pap smear test” and replace with “conventional cytology (Pap test)”

Response: Thank you for your comment. The ‘Pap smear test’ has been replaced with ‘conventional cytology (Pap test)’ (P5L84).

L85, L95 replace p16 with its full name, eventually in bracket p16.

Response: Thank you for your comment. ‘Cyclin-dependent kinase inhibitor’ followed by p16 in bracket has been added (P2L33-34, P6L108). In addition, ‘cyclin-dependent kinase inhibitor’ has been deleted with p16 written without brackets.

L109: replace “save” by “serve”

Response: Thank you for your comment. The sentence that has the word ‘serve’ has been accommodated in the revised goal of study “…. usefulness of p16 and TOP2A as potential biomarkers in dysplastic and malignant alteration of cervical epithelium by analyzing a series of benign, precancerous and cancerous cervical lesions …...” (P7L130-134).

L254: .... and TOP2A expressions among (Table 2). Finish the sentence.

Response: Thank you for your comment. The sentence has been completed to include ‘in the studied population’ (P16L273-274).

L255 replace “Also,” with “In addition,”

Response: Thank you for your comment. The word ‘Also’ has been replaced with ‘In addition’.

Lines 244, 251 and elsewhere: "among the studied women" replace by "in the studied population"

Response: Thank you for your good comment. The words ‘among the studied women’ and/or ‘among studied women’ have been replaced with ‘in the studied population’ throughout the whole manuscript.

L249 ? Error! Reference source not found.

Response: Thank you for your comment. The error associated with document formatting has been corrected.

L250 delete “Nevertheless,”

Response: Thank you for your comment. The word ‘Nevertheless’ has been deleted.

L285 “Overall, our findings suggest that p16 and TOP2A biomarkers could be associated with the development of cervical cancer lesions.” This statement is irrelevant as both biomarkers are commercially available as cervical cancer biomarkers. So, please rephrase your conclusion.

Response: Thank you for your good comment. We have rephrased the sentence, “Overall, our findings suggest that p16 and TOP2A biomarkers could be associated with the development of cervical cancer lesions” to “Overall, our findings support the association of p16 and TOP2A biomarkers in the development of cervical cancer lesions” (P18L302-303).

Replace everywhere “100.0% and 0.0%” by “100% and 0%”

Response: Thank you for your comment. All values with ‘100.0%’ and ‘0.0%’ have been replaced by ‘100%’ and ‘0.0%’, respectively throughout the manuscript.

Table 2 delete a double bracket: 1 (14.3))

Response: Thank you for your comment. A second “closing” bracket has been deleted.

**Reviewer #3**

Reviewer #3: The authors have notably improved the manuscript compared to the first version, considering the comments of the reviewers. Although the study of these proteins as biomarkers in cervical cancer is not new, the data are important due to their application in the study population. The article could be published in its current form.

Response: Thank you for very constructive comments to improve our manuscript.

Minor revisions

In Table 5, the authors must mention which variable they used to adjust the ORs; Were the ORs adjusted for age?

Response: Thank you for good comment. The odd ratios were adjusted for age. Table 5 has been revised to show AOR which stands for ‘age-adjusted odds ratio’. In addition, the strength of associations between p16 and TOP2A biomarkers in differentiating cancerous cervical lesions from benign and precancerous cervical lesions is described in the context of age-adjusted odds ratio (P18L297-303).
